# Pathogenic mechanism of second hand smoke induced inflammation and COPD

**DOI:** 10.3389/fphys.2012.00348

**Published:** 2012-08-28

**Authors:** Rahel L. Birru, Y. Peter Di

**Affiliations:** Department of Environmental and Occupational Health, University of PittsburghPittsburgh, PA, USA

**Keywords:** second hand smoke, inflammation, COPD, immunity, cancer

## Abstract

Second hand smoke (SHS) introduces thousands of toxic chemicals into the lung, including carcinogens and oxidants, which cause direct airway epithelium tissue destruction. It can also illicit indirect damage through its effect on signaling pathways related to tissue cell repair and by the abnormal induction of inflammation into the lung. After repeated exposure to SHS, these symptoms can lead to the development of pulmonary inflammatory disorders, including chronic obstructive pulmonary disease (COPD). COPD is a severe pulmonary disease characterized by chronic inflammation and irreversible tissue destruction. There is no causal cure, as the mechanism behind the development and progression of the disease is still unknown. Recent discoveries implicate genetic predisposition associated with inflammatory response contributed to the development of COPD, linked to irregular innate and adaptive immunity, as well as a risk factor for cancer. The use of animal models for both cigarette smoke (CS) and SHS associated *in vivo* experiments has been crucial in elucidating the pathogenic mechanisms and genetic components involved in inflammation-related development of COPD.

## Classification of SHS

Second hand smoke (SHS) is classified as exposure to sidestream smoke, produced directly by tobacco containing products (cigarettes, cigars, and pipes), or mainstream smoke, exhaled by smokers. The US Environmental Protection Agency determined in 1986 that SHS is a cause of lung cancer development, responsible for approximately 3000 lung cancer deaths annually (Jinot and Bayard, 1994; IARC, 2004; Talhout et al., 2011). In 2010, there were 10.3% adult smokers worldwide, or 45.3 million people, so the frequency of SHS exposure can be extensive (King et al., 2011). Around 10–25% of lung cancers are diagnosed in nonsmokers, who are considered to have smoked less than 100 cigarettes in their lifetime (Couraud et al., 2012). Evidence for the toxicity of SHS was found in non-smoking individuals with spouses who smoked cigarettes, who displayed elevated risks for lung cancer, heart disease, and respiratory disorders (HHS, 2006; Sebelius, 2011).

The burning tip of a cigarette is hot enough to allow for the release of tobacco smoke (TS) components into a gas and particulate vapor that is easily absorbed into the lung (Pappas, 2011; Talhout et al., 2011). This vapor rapidly enters the lower airways of the human lung, and eventually the circulatory and lymphatic systems (IARC, 2004; Baker, 2006). While tobacco is comprised of more than 5000 constituents, TS contains roughly 2800 molecules not found in tobacco, including reactive oxygen species (ROS) and nitric oxides (Baker, 2006). This indicates that the combustion, pyrolysis, and prosynthetic reactions during the flaming of the tobacco product are what create the components of TS (Baker, 2006). Approximately 250 carcinogenic and noxious chemicals have been measured in both sidestream and mainstream smoke (HHS, 2006). Mainstream smoke is generated at high temperatures in the presence of oxygen drawn through the column of a smoking apparatus, resulting in larger particles than sidestream smoke (HHS, 2006). Sidestream smoke is generated at lower temperatures in an oxygen-poor environment, with higher concentrations of ammonia, nitric oxides, and carcinogens (HHS, 2006). While all forms of environmental TS exposure have been shown to cause genetic damage, the detriments of SHS to a person vary based on proximity to source of smoke, time, and environment.

## SHS-induced inflammation and COPD

In response to SHS exposure, there is enhanced recruitment of inflammatory cells to the lung, particularly neutrophils and macrophages (Rennard et al., 2006). Short-term exposure to SHS does not result in a notable difference in inflammation in humans, though endothelial function deteriorates (Bonetti et al., 2011). Long-term TS exposure in mice leads to significantly increased inflammation, as measured by the influx of alveolar macrophages, neutrophils, and antioxidant enzymes (Bezerra et al., 2011). TS can also directly bind to DNA to effect the expression of genes related to inflammation. Sekhon et al. determined that nicotine can enter the placenta and directly interact with nicotine receptors on non-neuronal cells of the fetus (Sekhon et al., 1999). They also found that nicotine exposure leads to the enhancement of elastin and collagen type I and III mRNA expression, as well as airway wall expansion in the fetal lung (Sekhon et al., 2002).

SHS increases the incidence and severity of respiratory infections and disorders in humans (Jinot and Bayard, 1994; HHS, 2006; Sebelius, 2011). Exposure to TS introduces thousands of xenobiotics to the lung, and can lead to a persistent inflammatory response in the small airways and alveoli. This is the foundation for the development of pulmonary inflammatory disorders, such as COPD. COPD is a progressive and irreversible airflow obstructive disease of the lung and the third leading cause of death in the US. Of patients who are diagnosed with lung cancer, 40–70% of patients have COPD (Young et al., 2009). Chronic bronchitis, characterized by a consistent cough with mucus secretion, and emphysema, characterized by the destruction of airway epithelium and thickening of airway walls, is the distinct phenotypes that define COPD, though they can occur concurrently. Manifestation of COPD is a result of an interaction of TS exposure with other toxic environmental exposures, genetic factors, and unresolved childhood respiratory infections (Decramer et al., 2012). While TS is the main risk factor for COPD, only 20% of smokers develop COPD, suggesting a genetic predisposition (Young et al., 2009). Evidence for this includes the discovery of the genetic variants and mutations associated with TS-induced inflammation and COPD (Gwilt et al., 2007; Guo et al., 2012; Hunt and Tuder, 2012). These polymorphisms and mutations may be responsible for the exacerbation of inflammatory symptoms, resulting in COPD and lung cancer development (Young et al., 2009).

One of the most damaging effects of TS is oxidative damage, which promotes COPD development (Decramer et al., 2012). SHS contains >10^16^ free radicals per cigarette (Barcelo et al., 2008), comprising of ROS and peroxides (Baker, 2006). When introduced to the lung, an imbalance of oxidant and antioxidants, which protect against free radicals, occurs and results in oxidative stress (HHS, 2006). Oxidative stress induces direct airway epithelial damage, as well as indirect damage by altering signaling pathways. These pathways are related to cell proliferation, differentiation, and proinflammatory cytokines and chemokines through the upregulation of the transcription factors nuclear factor-κB (NF-κB) and activator-protein 1 (AP-1) (MacNee, 2001; HHS, 2006). Oxidative stress also leads to the oxidation of DNA, lipids, and proteins, resulting in lung injury and the production of secondary ROS (MacNee, 2001). Additionally, it can prevent repair processes in the damaged epithelium through inhibition or damage to surfactant and antiproteases, which leads to the development of fibrosis (MacNee, 2001; Decramer et al., 2012). Howard et al. developed a short-term SHS rat model and found considerable DNA damage in several tissues, measured by the presence of 8-hydroxy-2′-deoxyguanosine (8-OHdG), a major product of DNA oxidation (Howard et al., 1998). Chiang et al. found that 8-OHdG levels in human plasma increases with SHS exposure in a dose-dependent manor (Chiang et al., 2012).

Both the innate and adaptive immune responses play a role in the pathogenesis of COPD. In response to SHS, the innate immune response is triggered, resulting in inflammatory cell infiltration, mainly neutrophils and macrophages, to the mucosa and submucosa glands of the airway epithelium (van Antwerpen et al., 1995; MacNee, 2001; Decramer et al., 2012). Neutrophils and macrophages release neutrophil elastase and macrophage metalloproteases, respectively, along with pro-apoptotic factors to combat toxins and prevent the spread of injury. Accumulation of activated inflammatory cells from repeated SHS exposure reduces their usefulness, resulting in tissue damage and oxidative stress (Bosken et al., 1991; Rennard et al., 2006). This exacerbates TS-induced airway destruction, fibrosis, and remodeling, which are the basis for the development of inflammatory disorders (Bosken et al., 1991; Rennard et al., 2006).

TS can enhance the damaging phenotype of inflammatory cells. In study participants exposed to 3 hours of sidestream smoke, there was an average of 71% more reactive oxidants released by neutrophils (Anderson et al., 1991). Furthermore, activated polymorphonuclear cells are delayed in the lung microvessels by TS, allowing for enhanced tissue destruction (Klut et al., 1993). A positive correlation has been found with higher numbers of neutrophils in the circulating blood and reduced airway function, measured by spirometric levels (FEV1), in smoker lungs (van Antwerpen et al., 1995).

Dendritic cells are the link between the innate and adaptive immunity. If the innate immune response is unable to control the damage by TS, the recruited inflammatory cells, cytokines, chemokines, antigens, and other factors can induce dendritic cells to migrate to the lymphnodes for activation and differentiation (Cosio, 2004). Dendritic cells interact with T-cells and B-cells to instigate and shape the adaptive immune response. Naïve T-cells differentiate into several subsets, including T-helper 1 (Th1), T-Helper 2 (Th2), T-helper 17 (Th17), and regulatory T cells (Treg). These are distinct in the T-cell factors and cytokines they activate. The differentiation is largely dependent on the local inflammatory environment and the strength of the T cell receptor with the antigen (Zhou et al., 2009). The characteristic of the T-cells in disease manifestation and progression is important to consider, because the imbalance of T-cell populations can lead to irregular and severe inflammatory responses. Further analysis into the inflammatory microenvironment of the COPD lung has led to the discovery that the Th1 (Grumelli et al., 2004; Lee et al., 2007) and Th17 (Vargas-Rojas et al., 2011) subsets are particularly high in the COPD lung, with Th17 cells conceivably mediating the Th1 activity (Alcorn et al., 2010; Vanaudenaerde et al., 2011). Chen et al. exposed wild-type and IL-17Ra deficient mice to sidestream smoke for 6 months and found that the deficient mice developed significantly less tissue emphysema and airspace enlargement (Chen et al., 2011).

In addition, Tregs are absent in the bronchoalveolar lavage (BAL) fluid and blood of COPD patients (Lee et al., 2007; Barcelo et al., 2008), while smokers without COPD show an upregulation of this subtype (Barcelo et al., 2008). Tregs are critical in containing the immune response and maintaining tolerance to self-antigens. Therefore, without Treg regulation, continual exposure to TS can lead to an overpowering pro-inflammatory response mediated by Th1 and Th17 lymphocytes, resulting in the severe airway damage characterized by COPD.

B-cells have also been found to be upregulated in TS-driven emphysema patients. B-cell follicles were found in the bronchial walls and parenchyma of these patients and increased over time, which correlated with progressive airspace enlargement (van der Strate et al., 2006). While exposure to TS illicits airway damage and subsequent release of antigens by the innate immune system in all lungs, not all people react to the antigens and have resulting B- and T-cell differentiation, which explains why only a percentage of smokers develop COPD (Cosio et al., 2009). There is also a variation in the degree in which people react to the antigens, which explains the deviation in severity of the disease (Cosio et al., 2009).

Of growing interest is the hypothesis that COPD is linked to autoimmunity. Reduced levels of Tregs are an indication of autoimmunity (Shevach, 2000). Also, in order for T-cells to migrate to the lung, they must be activated by antigens (self or modified-self) (Cosio, 2004). Lee et al. discovered that antibodies toward elastin, a self-antigen, were significantly increased in emphysema patients (Lee et al., 2007). They propose TS-exposure leads to proteolytic-induced cleavage of elastin, resulting in fragments that generate T- and B-cell immunity against elastin (Lee et al., 2007). Kirkham et al. propose that chronic oxidative stress in COPD induces carbonyl-modification of self-proteins, creating neoantigens that are targeted by the immune system. In support of this hypothesis, they found increased antibody titer against carbonyl-modified self-proteins in COPD patients versus control subjects (Kirkham et al., 2011). Additionally, the persistence of COPD symptoms after smoking cessation indicates that T- and B-cells are recruited in response to self-antigens (Motz et al., 2008; Cosio et al., 2009).

## Inflammation and cancer

The enhanced inflammatory cell environment of the lung from exposure to TS can promote the development of mutated cells into malignant cells, eventually resulting in tumor formation and progression. While acute inflammation inhibits tumor growth, long-term inflammation promotes tumor enlargement and metastasis. Because TS can compromise alveolar repair mechanisms, such as chemotaxis, apoptosis, and matrix restoration, these malignant cells can develop into tumors and metastasize (Rennard et al., 2006). Jinushi et al. generated genetically modified mice study the relationship of chronic inflammation and lung cancer by simulating defects in apoptotic cell clearance, autoreactive Th17, and increased vulnerability to infection (Jinushi et al., 2007). These mice developed chronic pulmonary inflammation and lung adenocarcinomas, as well as increased mortality (Jinushi et al., 2007).

The main link between chronic inflammation and oncogenesis is considered to be TNF-α mediated upregulation of NF-κB, which induces anti-apoptotic and proliferative effects. TNF-α has a seemingly contradictory role of stimulating apoptosis through activation of caspase 8, while simultaneously activating NF-κB, which protects cells from pro-apoptotic stimuli. NF-κB is a transcription factor which plays an integral role in the immune response to infection. It is activated by cellular signals resulting from stimuli such as necrotic cells, cytokines, and ROS. Once activated, NF-κB translocates into the nucleus to bind to DNA, activating hundreds of different genes encoding proteins related to the immune response, inflammation, and cell growth. In an environment of pre-malignant cells due to environmental exposures like SHS, continual NF-κB activation will support tumor development and progression by inhibiting apoptosis while activating cell proliferation, metastasis, and survival through the products of genes it regulates (Karin et al., 2002; Luo et al., 2004; Philip et al., 2004; Karin, 2006).

In addition to TNF-α, there are other molecular pathways implicated in upregulating NF-κ B expression and other transcription factors (TFs). Zhao et al. used protein and DNA arrays to examine potential upstream signaling pathways responsible for TS-induced TF activation. By exposing cells to TS, they examined 244 different TFs. TS significantly regulates at least 20 TFs including NF-κ B, which may be involved in tumorigenesis and cell cycle regulation, activated primarily by MAPK signaling pathways (Zhao et al., 2007). These results indicate that MAPK signaling is also essential in TS-induced NF-κ B activation and subsequent inflammatory gene expression.

The long-term use of anti-inflammatory agents have been linked to decreased cancer incidence, indicating inflammation as a contributor to cancer development (Dougan et al., 2011). Witschi et al. exposed mice both mainstream and sidestream smoke for 5 months, followed by a 4 month recovery period (Witschi et al., 2005). When mice were introduced to dexamethasone, an anti-inflammatory and immunosuppressant glucocorticoid steroid drug, for 4 months, the lung tumor multiplicity decreased by 64% compared to control mice (Witschi et al., 2005).

COPD is believed to be an independent risk factor for lung cancer. Prevalence of COPD in lung cancer cases is six-fold higher than in smokers without lung cancer (Young et al., 2009). Because chronic airway inflammation is a risk factor for COPD and is related to the increase of human cancers, it is hypothesized that COPD and lung cancer may share chronic inflammation as a common pathogenic mechanism (Young et al., 2009).

## Use of animal models

Animal models continue to be crucial in determining the genetic factors underlying SHS and COPD (Table 1). When considering mouse models for experimental use, the choice of strain is critical in studies related to both SHS and COPD as there are strain-related differences in the metabolism of TS as well as the inflammatory cell composition and magnitude. Vecchio et al. examined this issue by comparing C57BL/6J and Institute of Cancer Research (ICR) mice post-cigarette smoke extract exposure (Vecchio et al., 2010). They found that alveolar macrophages from C57BL/6J mice produced higher levels of ROS, NF-κ B, and proinflammatory cytokines (Vecchio et al., 2010). They hypothesize that the higher pro-inflammatory response in C57BL/6J versus ISC mice is due to higher oxidative stress in this strain, leading to increased activation of NF-κ B. This may describe the differences in susceptibility of the different strains of mice (Vecchio et al., 2010). Cavarra et al. found that after acute TS exposure, DBA/2 and C57BL/6J mice had decreased antioxidant defenses, measured in bronchoalveolar lavage fluid, while ICR mice had increased antioxidants (Cavarra et al., 2001). After chronic exposure to TS for 7 months, they found that DBA/2 and C57BL/6J mice are more likely to develop emphysema and decreased lung elastin levels, while ICR mice did not develop these phenotypes (Cavarra et al., 2001). Tsuji et al. compared CS-exposure in AKR/J and C57BL/6J mice and found that C57BL/6J mice inhaled higher amounts of smoke and more severe respiratory lesions, while AKR/J mice had higher inflammatory cytokine levels (Tsuji et al., 2011).

**Table 1:** **Factors that influence SHS-induced COPD development and progression**.

**Exposure**	**Factor**	**Role in COPD development**	**Animal model**	**Strain background**	**References**
Lipopolysacharide	Acetylcholine	Airway remodeling and emphysema	Guinea pig	Dunkin Hartley	Pera et al., 2011
Cigarette smoke Extract	Akt serine/threonine protein kinase (Akt)	Reduces cytotoxicity of TS, TS-exposure causes ubiquination of Akt	Rat	Lewis	Kim et al., 2011
Cigarette smoke	Caspase 1 (Casp1)	Inflammatory cell influx through IL-1β/IL-18	Mouse	C57BL/6	Churg et al., 2009
Cigarette smoke	C-Jun/Activator protein 1 (AP-1)	Regulates inflammation after long-term SHS exposure, restrains emphysema symptoms	Mouse	C57BL/6 × 129SVJ	Reddy et al., 2012
Cigarette smoke	Clara cell 10 kDa (Ccsp)	Protects the airway epithelium, TS exposure causes metaplasia of clara cells	Mouse	BALB/c	Cuzic et al., 2012
Cigarette smoke	Chemokine (C-X3-C) receptor 1 (Cx3cr1)	Required for IL-6 and TNF-α production by phagocytes; development of emphysema phenotype	Mouse	C57BL/6	Xiong et al., 2011
Cigarette smoke	Early growth response-1 (Egr-1)	Promotes autophagy and apoptosis in early stages of COPD	Mouse	C57BL/6	Chen et al., 2008
Cigarette smoke	endothelial monocyte-activating protein 2 (EmapII)	Inducing apoptosis through caspase 3, macrophage influx, emphysema phenotype	Mouse	C57BL/6	Clauss et al., 2011
Cigarette smoke	Extracellular signal-regulated kinase 1/2 (Erk 1/2)	Airway mucus hypersecretion	Rat	Sprague-Dawley	Xiao et al., 2011
Cigarette smoke	Extracellular superoxide dismutase (Ecsod)	Reduces TS-induced oxidative stress	Mouse	C57BL/6	Tollefson et al., 2010
Cigarette smoke	Forkhead box O3 (Foxo3)	Regulates inflammation, antioxidant genes; downregulated in COPD	Mouse	FVBx129S6	Hwang et al., 2011
Cigarette smoke	Granulocyte/Macrophage colony–stimulating factor (Gm-CSf)	Initiation of inflammatory cell influx	Mouse	BALB/c	Vlahos et al., 2010
Cigarette smoke	IFN regulatory factor (Irf7)	Inhibited in COPD lung, dampens proinflammatory cytokines in lung dendritic cells	Mouse	C57BL/6	Shan et al., 2012
Overexpression of IL-11 through transgenic mouse model	Interleukin 11 (Il-11)	Emphysema phenotype, airway remodeling and fibrosis	Mouse	Not reported	Kuhn et al., 2000
Cigarette smoke extract	Interleukin 17 Receptor A (Il-17RA)	Induces matrix metalloproteinase-12 (MMP-12) and CCl2, required for emphysema development	Mouse	C57BL/6	Chen et al., 2011
Cigarette smoke	Interleukin 1 Receptor, Type 1 (Il1R1)	Critical in initiation of neutrophilic inflammatory response to short-term TS	Mouse	C57BL/6	Doz et al., 2008
Cigarette smoke	Interleukin 1 alpha (Il-1α)	Critical in the initiation of the neutrophilic inflammatory response to TS	Mouse	BALB/c	Botelho et al., 2011
Overexpression of IL1-β through transgenic mouse model	Interleukin 1 beta (Il1-β)	Macrophage and neutrophil influx, emphysema phenotype	Mouse	Not reported	Lappalainen et al., 2005
Overexpression of IL-6 through transgenic mouse model	Interleukin 6 (Il-6)	Emphysema phenotype, airway remodeling and fibrosis	Mouse	Not reported	Kuhn et al., 2000
Cigarette smoke	Mucin-5ac (Muc5ac)	Mucus secretion in response to pro-inflammatory cytokines	Rat	Sprague-Dawley	Xiao et al., 2011
Cigarette smoke	NADPH oxidase (Nox)	Highly expressed in neutrophils, source of oxidative stress	Mouse	C57BL/6	Tollefson et al., 2010
Cigarette smoke	Osteopontin (Opn)	Th17 differentiation, emphysema phenotype	Mouse	C57BL/6	Shan et al., 2012
Cigarette smoke	P2X7	Neutrophil influx, caspase 1 activity, IL-1β	Mouse	C57BL/6	Eltom et al., 2011
Lipopolysacharide and cigarette smoke	Peroxisome proliferator-activated receptor-gamma (Pparγ)/PPARγ coactivator-1α (Pgc-1α)	Relieves oxidative stress; expression decreases with progression of COPD	Rat	Sprague-Dawley	Li et al., 2010
Cigarette smoke	Transforming growth factor beta (Tgf-β)	Airway remodeling, emphysema phenotype	Mouse	AKR/J, C57BL/6	Podowski et al., 2012
Cigarette smoke	Toll-like receptor 4 (Tlr4)	Critical in the initiation of the neutrophilic inflammatory response to TS short-term (61, 63); Role is diminished in chronic TS-exposure model (61)	Mouse	C3H/HeJ (Maes), C57BL/6J (Doz)	Maes et al., 2006; Doz et al., 2008
Cigarette smoke	Toll-like receptor 4 (Tlr4)	Induces Metalloproteinase-1 (MMP-1), required for emphysema development	Mouse, Rabbit	C57BL/CBA, New Zealand White	Geraghty et al., 2011
Cigarette smoke	Tumor necrosis factor-alpha (Tnf-α)	Inflammatory cell influx, chronic inflammation, emphysema phenotype	Mouse	C57BL/6	Churg et al., 2004
Knock-out genetic model	Vascular endothelial growth factor (Vegf)	Preventative role in emphysema	Mouse	C57BL/6	Tang et al., 2004

Listed in alphabetical order are proteins, receptors, and other factors which have been discovered in animal models that influence SHS-induced COPD. Their degree of influence in vivo could vary based on the experimental design; therefore the exposure models and mouse strains are also included in the table.

**Figure 1:**
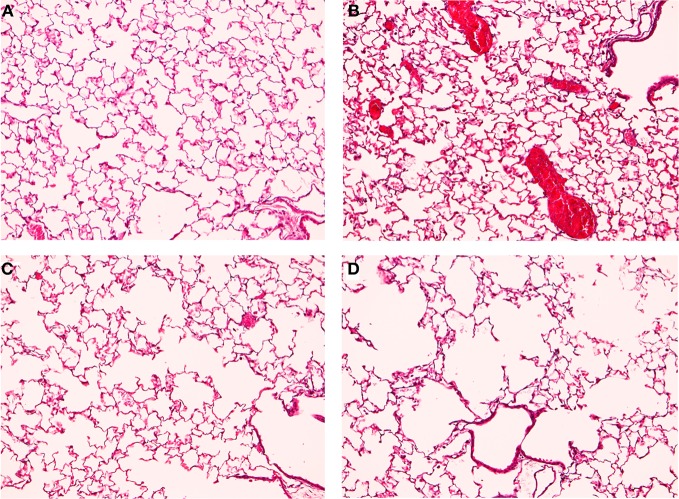
**TS and LPS treated mice have enhanced inflammation and alveolar space enlargement.** Mice were exposed to filtered air **(A,B)** or TS **(C,D)** generated by Kentucky Research Cigarettes for 6 months. Inflammation was further induced by intranasal LPS instillation **(B,D)**, with saline as a control **(A,C)**. Lung histology was analyzed by staining lung sections with hematoxylin and eosin. Results shown are representative images for each treatment at 10X magnification. TS-exposed mice displayed alveolar space enlargement **(C)** compared to filtered air exposure **(A)**. LPS stimulated inflammatory cell influx **(B)** and enhanced the alveolar space enlargement induced by TS **(D)** relative to TS-only exposure **(C)**.

Our lab developed a mouse model to simulate the development of COPD and SHS (Birru et al., 2012). We separated mice into four treatment groups: filtered-air control, lipopolysacharide (LPS) to stimulate inflammation, CS, and LPS combined with CS. Mice were sacrificed after 6 months of weekly LPS and daily CS exposure. The inflammatory response, alveolar space enlargement, and lung tumor incidence were assessed. In the LPS only group, mice displayed increased inflammation, but no alveolar space enlargement (Figure 1). In the LPS and CS group, mice displayed enhanced inflammation and alveolar space enlargement compared to the CS only group (Figure 1). No groups developed tumors in this exposure model at the duration examined. Our results indicate that chronic inflammation enhances emphysema-like alveolar space enlargement.

We also developed an animal exposure paradigm using components of TS to determine the role of inflammation in the development and progression of tumor formation (Keohavong et al., 2011). We used LPS to incite inflammation and nicotine-derived nitrosamine (NNK) for tumorigenesis (14). Mice were assigned into four different treatment groups: saline control, LPS only, NNK only, and LPS combined with NNK for 4 months. The saline and LPS only groups had no tumor development, but there was a six-fold increase in tumor numbers in the LPS and NNK group compared to the NNK only group. The LPS only and LPS with NNK groups displayed significantly elevated inflammation compared to the saline and NNK only groups. Our results indicate that repeated exposure to inflammation enhances the progression of TS carcinogen-induced lung tumorigenesis.

## Conclusions

SHS exposure is detrimental to the lung, resulting in lung destruction through the introduction of toxic chemicals to the lung and oxidants, as well as the inhibiting the repair pathways of the lung. Continued SHS exposure can also lead to the development of inflammation, which worsen COPD, due to the abnormal polarization of T- and B-cell differentiation. The enhanced inflammatory environment of the lung can also promote tumor initiation and progression of malignant cells through the activation of transcription factors that promote cell proliferation and inhibit apoptosis. Both environmental factors and genetic components underlying COPD continue to be uncovered, and will be crucial in developing useful treatments for the disease. Animal models for SHS-driven COPD studies will continue to play an essential role for this objective.

### Conflict of interest statement

The authors declare that the research was conducted in the absence of any commercial or financial relationships that could be construed as a potential conflict of interest.

## References

[B1] Alcorn J. F. Crowe C. R. Kolls J. K. (2010). TH17 cells in asthma and COPD. Annu. Rev. Physiol. 72, 495–516 10.1146/annurev-physiol-021909-135926 20148686

[B2] Anderson R. Theron A. J. Richards G. A. Myer M. S. van Rensburg A. J. (1991). Passive smoking by humans sensitizes circulating neutrophils. Am. Rev. Respir. Dis. 144, 570–574 1892296 10.1164/ajrccm/144.3_Pt_1.570

[B3] Baker R. R. (2006). Smoke generation inside a burning cigarette: modifying combustion to develop cigarettes that may be less hazardous to health. Prog. Energ. Combust. Sci. 32, 373–385

[B4] Barcelo B. Pons J. Ferrer J. M. Sauleda J. Fuster A. Agusti A. G. (2008). Phenotypic characterisation of T-lymphocytes in COPD: abnormal CD4+CD25+ regulatory T-lymphocyte response to tobacco smoking. Eur. Respir. J. 31, 555–562 10.1183/09031936.00010407 18057064

[B5] Bezerra F. S. Valenca S. S. Pires K. M. Lanzetti M. Pimenta W. A. Schmidt A. C. Porto L. C. Zin W. A. (2011). Long-term exposure to cigarette smoke impairs lung function and increases HMGB-1 expression in mice. Respir. Physiol. Neurobiol. 177, 120–126 10.1016/j.resp.2011.03.023 21457800

[B6] Birru R. Kahkonon B. Di Y. P. (2012). Chronic inflammation in the pathogenesis of COPD and lung cancer. Proc. Am. Thorac. Soc. 9, 81

[B7] Bonetti P. O. Lardi E. Geissmann C. Kuhn M. U. Bruesch H. Reinhart W. H. (2011). Effect of brief secondhand smoke exposure on endothelial function and circulating markers of inflammation. Atherosclerosis 215, 218–222 10.1016/j.atherosclerosis.2010.12.011 21215401

[B8] Bosken C. H. Doerschuk C. M. English D. Hogg J. C. (1991). Neutrophil kinetics during active cigarette smoking in rabbits. J. Appl. Physiol. 71, 630–637 1657860 10.1152/jappl.1991.71.2.630

[B9] Botelho F. M. Bauer C. M. Finch D. Nikota J. K. Zavitz C. C. Kelly A. Lambert K. N. Piper S. Foster M. L. Goldring J. J. Wedzicha J. A. Bassett J. Bramson J. Iwakura Y. Sleeman M. Kolbeck R. Coyle A. J. Humbles A. A. Stampfli M. R. (2011). IL-1alpha/IL-1R1 expression in chronic obstructive pulmonary disease and mechanistic relevance to smoke-induced neutrophilia in mice. PLoS ONE 6:e28457 10.1371/journal.pone.0028457 22163019 PMC3232226

[B10] Cavarra E. Bartalesi B. Lucattelli M. Fineschi S. Lunghi B. Gambelli F. Ortiz L. A. Martorana P. A. Lungarella G. (2001). Effects of cigarette smoke in mice with different levels of alpha(1)-proteinase inhibitor and sensitivity to oxidants. Am. J. Respir. Crit. Care Med. 164, 886–890 11549550 10.1164/ajrccm.164.5.2010032

[B11] Chen K. Pociask D. A. Mcaleer J. P. Chan Y. R. Alcorn J. F. Kreindler J. L. Keyser M. R. Shapiro S. D. Houghton A. M. Kolls J. K. Zheng M. (2011). IL-17RA is required for CCL2 expression, macrophage recruitment, and emphysema in response to cigarette smoke. PLoS ONE 6:e20333 10.1371/journal.pone.0020333 21647421 PMC3103542

[B12] Chen Z. H. Kim H. P. Sciurba F. C. Lee S. J. Feghali-Bostwick C. Stolz D. B. Dhir R. Landreneau R. J. Schuchert M. J. Yousem S. A. Nakahira K. Pilewski J. M. Lee J. S. Zhang Y. Ryter S. W. Choi A. M. (2008). Egr-1 regulates autophagy in cigarette smoke-induced chronic obstructive pulmonary disease. PLoS ONE 3:e3316 10.1371/journal.pone.0003316 18830406 PMC2552992

[B13] Chiang H. C. Huang Y. K. Chen P. F. Chang C. C. Wang C. J. Lin P. Lee H. L. (2012). 4-(Methylnitrosamino)-1-(3-pyridyl)-1-butanone is correlated with 8-hydroxy-2'-deoxyguanosine in humans after exposure to environmental tobacco smoke. Sci. Total Environ. 414, 134–139 10.1016/j.scitotenv.2011.11.039 22138374

[B14] Churg A. Wang R. D. Tai H. Wang X. Xie C. Wright J. L. (2004). Tumor necrosis factor-alpha drives 70% of cigarette smoke-induced emphysema in the mouse. Am. J. Respir. Crit. Care Med. 170, 492–498 10.1164/rccm.200404-511OC 15184206

[B15] Churg A. Zhou S. Wang X. Wang R. Wright J. L. (2009). The role of interleukin-1beta in murine cigarette smoke-induced emphysema and small airway remodeling. Am. J. Respir. Cell Mol. Biol. 40, 482–490 10.1165/rcmb.2008-0038OC 18931327

[B16] Clauss M. Voswinckel R. Rajashekhar G. Sigua N. L. Fehrenbach H. Rush N. I. Schweitzer K. S. Yildirim A. O. Kamocki K. Fisher A. J. Gu Y. Safadi B. Nikam S. Hubbard W. C. Tuder R. M. Twigg H. L. 3rd Presson R. G. Sethi S. Petrache I. (2011). Lung endothelial monocyte-activating protein 2 is a mediator of cigarette smoke-induced emphysema in mice. J. Clin. Invest. 121, 2470–2479 10.1172/JCI43881 21576822 PMC3104742

[B17] Cosio M. G. (2004). Autoimmunity, T-cells and STAT-4 in the pathogenesis of chronic obstructive pulmonary disease. Eur. Respir. J. 24, 3–5 15293596 10.1183/09031936.04.00043104

[B18] Cosio M. G. Saetta M. Agusti A. (2009). Immunologic aspects of chronic obstructive pulmonary disease. N. Engl. J. Med. 360, 2445–2454 10.1056/NEJMra0804752 19494220

[B19] Couraud S. Zalcman G. Milleron B. Morin F. Souquet P. J. (2012). Lung cancer in never smokers—A review. Eur. J. Cancer 48, 1299–1311 10.1016/j.ejca.2012.03.007 22464348

[B20] Cuzic S. Bosnar M. Dominis Kramaric M. Ferencic Z. Markovic D. Glojnaric I. Erakovic Haber V. (2012). Claudin-3 and clara cell 10 kDa protein as early signals of cigarette smoke-induced epithelial injury along alveolar ducts. Toxicol. Pathol. PMID: 22659244. [Epub ahead of print]. 10.1177/0192623312448937 22659244

[B21] Decramer M. Janssens W. Miravitlles M. (2012). Chronic obstructive pulmonary disease. Lancet 379, 1341–1351 10.1016/S0140-6736(11)60968-9 22314182 PMC7172377

[B22] Dougan M. Li D. Neuberg D. Mihm M. Googe P. Wong K. K. Dranoff G. (2011). A dual role for the immune response in a mouse model of inflammation-associated lung cancer. J. Clin. Invest. 121, 2436–2446 10.1172/JCI44796 21537082 PMC3104747

[B23] Doz E. Noulin N. Boichot E. Guenon I. Fick L. Le Bert M. Lagente V. Ryffel B. Schnyder B. Quesniaux V. F. Couillin I. (2008). Cigarette smoke-induced pulmonary inflammation is TLR4/MyD88 and IL-1R1/MyD88 signaling dependent. J. Immunol. 180, 1169–1178 18178857 10.4049/jimmunol.180.2.1169

[B24] Eltom S. Stevenson C. S. Rastrick J. Dale N. Raemdonck K. Wong S. Catley M. C. Belvisi M. G. Birrell M. A. (2011). P2X7 receptor and caspase 1 activation are central to airway inflammation observed after exposure to tobacco smoke. PLoS ONE 6:e24097 10.1371/journal.pone.0024097 21915284 PMC3167831

[B25] Geraghty P. Dabo A. J. D'armiento J. (2011). TLR4 protein contributes to cigarette smoke-induced matrix metalloproteinase-1 (MMP-1) expression in chronic obstructive pulmonary disease. J. Biol. Chem. 286, 30211–30218 10.1074/jbc.M111.238824 21730072 PMC3191060

[B26] Grumelli S. Corry D. B. Song L. Z. Song L. Green L. Huh J. Hacken J. Espada R. Bag R. Lewis D. E. Kheradmand F. (2004). An immune basis for lung parenchymal destruction in chronic obstructive pulmonary disease and emphysema. PLoS Med. 1:e8 10.1371/journal.pmed.0010008 15526056 PMC523885

[B27] Guo Y. Gong Y. Shi G. Yang K. Pan C. Li M. Li Q. Cheng Q. Dai R. Fan L. Wan H. (2012). Single-nucleotide polymorphisms in the TSPYL-4 and NT5DC1 genes are associated with susceptibility to chronic obstructive pulmonary disease. Mol. Med. Report 6, 631–638 10.3892/mmr.2012.964 22736055

[B28] Gwilt C. R. Donnelly L. E. Rogers D. F. (2007). The non-neuronal cholinergic system in the airways: an unappreciated regulatory role in pulmonary inflammation? Pharmacol. Ther. 115, 208–222 10.1016/j.pharmthera.2007.05.007 17597218

[B50] HHS. (2006). The Health Consequences of Involuntary Exposure to Tobacco Smoke: A Report of the Surgeon General. Atlanta, GA: U.S. Department of Health and Human Services, Centers for Disease Control and Prevention, Coordinating Center for Health Promotion, National Center for Chronic Disease Prevention and Health Promotion, Office on Smoking and Health

[B29] Howard D. J. Briggs L. A. Pritsos C. A. (1998). Oxidative DNA damage in mouse heart, liver, and lung tissue due to acute side-stream tobacco smoke exposure. Arch. Biochem. Biophys. 352, 293–297 10.1006/abbi.1998.0605 9587419

[B30] Hunt J. M. Tuder R. (2012). Alpha 1 anti-trypsin: one protein, many functions. Curr. Mol. Med. 12, 827–835 22697349 10.2174/156652412801318755

[B31] Hwang J. W. Rajendrasozhan S. Yao H. Chung S. Sundar I. K. Huyck H. L. Pryhuber G. S. Kinnula V. L. Rahman I. (2011). FOXO3 deficiency leads to increased susceptibility to cigarette smoke-induced inflammation, airspace enlargement, and chronic obstructive pulmonary disease. J. Immunol. 187, 987–998 10.4049/jimmunol.1001861 21690325 PMC3131437

[B32] IARC. (2004). Tobacco smoke and involuntary smoking. IARC Monogr. Eval. Carcinog. Risks Hum. 83, 1–1438 15285078 PMC4781536

[B33] Jinot J. Bayard S. (1994). Respiratory health effects of passive smoking: EPA's weight-of-evidence analysis. J. Clin. Epidemiol. 47, 339–349 discussion: 351–333. 7730859 10.1016/0895-4356(94)90154-6

[B34] Jinushi M. Nakazaki Y. Dougan M. Carrasco D. R. Mihm M. Dranoff G. (2007). MFG-E8-mediated uptake of apoptotic cells by APCs links the pro- and antiinflammatory activities of GM-CSF. J. Clin. Invest. 117, 1902–1913 10.1172/JCI30966 17557120 PMC1884688

[B35] Karin M. (2006). Nuclear factor-kappaB in cancer development and progression. Nature 441, 431–436 10.1038/nature04870 16724054

[B36] Karin M. Cao Y. Greten F. R. Li Z. W. (2002). NF-kappaB in cancer: from innocent bystander to major culprit. Nat. Rev. Cancer 2, 301–310 10.1038/nrc780 12001991

[B37] Keohavong P. Kahkonen B. Kinchington E. Yin J. Jin J. Liu X. Siegfried J. M. Di Y. P. (2011). K-ras mutations in lung tumors from NNK-treated mice with lipopolysaccharide-elicited lung inflammation. Anticancer Res. 31, 2877–2882 21868532

[B38] Kim S. Y. Lee J. H. Huh J. W. Ro J. Y. Oh Y. M. Lee S. D. An S. Lee Y. S. (2011). Cigarette smoke induces Akt protein degradation by the ubiquitin-proteasome system. J. Biol. Chem. 286, 31932–31943 10.1074/jbc.M111.267633 21778238 PMC3173210

[B39] King B. Dube S. Kaufmann R. Shaw L. Pechacek T. (2011). Vital signs: current cigarette smoking among adults aged = 18 years-United States, 2005–2010 (Reprinted from MMWR 60, 1207–1212, 2011). JAMA 306, 1857–1860 21900875

[B40] Kirkham P. A. Caramori G. Casolari P. Papi A. A. Edwards M. Shamji B. Triantaphyllopoulos K. Hussain F. Pinart M. Khan Y. Heinemann L. Stevens L. Yeadon M. Barnes P. J. Chung K. F. Adcock I. M. (2011). Oxidative stress-induced antibodies to carbonyl-modified protein correlate with severity of chronic obstructive pulmonary disease. Am. J. Respir. Crit. Care Med. 184, 796–802 10.1164/rccm.201010-1605OC 21965015 PMC3398415

[B41] Klut M. E. Doerschuk C. M. Van Eeden S. F. Burns A. R. Hogg J. C. (1993). Activation of neutrophils within pulmonary microvessels of rabbits exposed to cigarette smoke. Am. J. Respir. Cell Mol. Biol. 9, 82–89 10.1165/ajrcmb/9.1.82 7687850

[B42] Kuhn C. 3rd Homer R. J. Zhu Z. Ward N. Flavell R. A. Geba G. P. Elias J. A. (2000). Airway hyperresponsiveness and airway obstruction in transgenic mice. Morphologic correlates in mice overexpressing interleukin (IL)-11 and IL-6 in the lung. Am. J. Respir. Cell Mol. Biol. 22, 289–295 10696065 10.1165/ajrcmb.22.3.3690

[B43] Lappalainen U. Whitsett J. A. Wert S. E. Tichelaar J. W. Bry K. (2005). Interleukin-1beta causes pulmonary inflammation, emphysema, and airway remodeling in the adult murine lung. Am. J. Respir. Cell Mol. Biol. 32, 311–318 10.1165/rcmb.2004-0309OC 15668323

[B44] Lee S. H. Goswami S. Grudo A. Song L. Z. Bandi V. Goodnight-White S. Green L. Hacken-Bitar J. Huh J. Bakaeen F. Coxson H. O. Cogswell S. Storness-Bliss C. Corry D. B. Kheradmand F. (2007). Antielastin autoimmunity in tobacco smoking-induced emphysema. Nat. Med. 13, 567–569 10.1038/nm1583 17450149

[B45] Li J. Dai A. Hu R. Zhu L. Tan S. (2010). Positive correlation between PPARgamma/PGC-1alpha and gamma-GCS in lungs of rats and patients with chronic obstructive pulmonary disease. Acta Biochim. Biophys. Sin. (Shanghai) 42, 603–614 10.1093/abbs/gmq071 20732852

[B46] Luo J. L. Maeda S. Hsu L. C. Yagita H. Karin M. (2004). Inhibition of NF-kappaB in cancer cells converts inflammation-induced tumor growth mediated by TNFalpha to TRAIL-mediated tumor regression. Cancer Cell 6, 297–305 10.1016/j.ccr.2004.08.012 15380520

[B47] MacNee W. (2001). Oxidative stress and lung inflammation in airways disease. Eur. J. Pharmacol. 429, 195–207 11698041 10.1016/s0014-2999(01)01320-6

[B48] Maes T. Bracke K. R. Vermaelen K. Y. Demedts I. K. Joos G. F. Pauwels R. A. Brusselle G. G. (2006). Murine TLR4 is implicated in cigarette smoke-induced pulmonary inflammation. Int. Arch. Allergy Immunol. 141, 354–368 10.1159/000095462 16940747

[B49] Motz G. T. Eppert B. L. Sun G. Wesselkamper S. C. Linke M. J. Deka R. Borchers M. T. (2008). Persistence of lung CD8 T cell oligoclonal expansions upon smoking cessation in a mouse model of cigarette smoke-induced emphysema. J. Immunol. 181, 8036–8043 19017996 10.4049/jimmunol.181.11.8036

[B51] Pappas R. S. (2011). Toxic elements in tobacco and in cigarette smoke: inflammation and sensitization. Metallomics 3, 1181–1198 10.1039/c1mt00066g 21799956 PMC4542087

[B52] Pera T. Zuidhof A. Valadas J. Smit M. Schoemaker R. G. Gosens R. Maarsingh H. Zaagsma J. Meurs H. (2011). Tiotropium inhibits pulmonary inflammation and remodelling in a guinea pig model of COPD. Eur. Respir. J. 38, 789–796 10.1183/09031936.00146610 21349917

[B53] Philip M. Rowley D. A. Schreiber H. (2004). Inflammation as a tumor promoter in cancer induction. Semin. Cancer Biol. 14, 433–439 10.1016/j.semcancer.2004.06.006 15489136

[B54] Podowski M. Calvi C. Metzger S. Misono K. Poonyagariyagorn H. Lopez-Mercado A. Ku T. Lauer T. Mcgrath-Morrow S. Berger A. Cheadle C. Tuder R. Dietz H. C. Mitzner W. Wise R. Neptune E. (2012). Angiotensin receptor blockade attenuates cigarette smoke-induced lung injury and rescues lung architecture in mice. J. Clin. Invest. 122, 229–240 10.1172/JCI46215 22182843 PMC3248282

[B55] Reddy N. M. Vegiraju S. Irving A. Paun B. C. Luzina I. G. Atamas S. P. Biswal S. Ana N. A. Mitzner W. Reddy S. P. (2012). Targeted deletion of Jun/AP-1 in alveolar epithelial cells causes progressive emphysema and worsens cigarette smoke-induced lung inflammation. Am. J. Pathol. 180, 562–574 10.1016/j.ajpath.2011.10.029 22265050 PMC3349866

[B56] Rennard S. I. Togo S. Holz O. (2006). Cigarette smoke inhibits alveolar repair: a mechanism for the development of emphysema. Proc. Am. Thorac. Soc. 3, 703–708 10.1513/pats.200605-121SF 17065377 PMC2647656

[B57] Sebelius K. (2011). NTP 12th report on carcinogens. Rep. Carcinog. 12, iii-499 21822324

[B58] Sekhon H. S. Jia Y. Raab R. Kuryatov A. Pankow J. F. Whitsett J. A. Lindstrom J. Spindel E. R. (1999). Prenatal nicotine increases pulmonary alpha7 nicotinic receptor expression and alters fetal lung development in monkeys. J. Clin. Invest. 103, 637–647 10.1172/JCI5232 10074480 PMC408124

[B59] Sekhon H. S. Keller J. A. Proskocil B. J. Martin E. L. Spindel E. R. (2002). Maternal nicotine exposure upregulates collagen gene expression in fetal monkey lung. Association with alpha7 nicotinic acetylcholine receptors. Am. J. Respir. Cell Mol. Biol. 26, 31–41 11751201 10.1165/ajrcmb.26.1.4170

[B60] Shan M. Yuan X. Song L. Z. Roberts L. Zarinkamar N. Seryshev A. Zhang Y. Hilsenbeck S. Chang S. H. Dong C. Corry D. B. Kheradmand F. (2012). Cigarette smoke induction of osteopontin (SPP1) mediates T(H)17 inflammation in human and experimental emphysema. Sci. Transl. Med. 4, 117ra119 10.1126/scitranslmed.3003041 22261033 PMC3956594

[B61] Shevach E. M. (2000). Regulatory T cells in autoimmmunity. Annu. Rev. Immunol. 18, 423–449 10.1146/annurev.immunol.18.1.423 10837065

[B62] Talhout R. Schulz T. Florek E. van Benthem J. Wester P. Opperhuizen A. (2011). Hazardous compounds in tobacco smoke. Int. J. Environ. Res. Public Health 8, 613–628 10.3390/ijerph8020613 21556207 PMC3084482

[B63] Tang K. Rossiter H. B. Wagner P. D. Breen E. C. (2004). Lung-targeted VEGF inactivation leads to an emphysema phenotype in mice. J. Appl. Physiol. 97, 1559–1566 discussion: 1549. 10.1152/japplphysiol.00221.2004 15208295

[B64] Tollefson A. K. Oberley-Deegan R. E. Butterfield K. T. Nicks M. E. Weaver M. R. Remigio L. K. Decsesznak J. Chu H. W. Bratton D. L. Riches D. W. Bowler R. P. (2010). Endogenous enzymes (NOX and ECSOD) regulate smoke-induced oxidative stress. Free Radic. Biol. Med. 49, 1937–1946 10.1016/j.freeradbiomed.2010.09.022 20887783 PMC3780970

[B65] Tsuji H. Fujimoto H. Matsuura D. Nishino T. Lee K. M. Renne R. Yoshimura H. (2011). Comparison of mouse strains and exposure conditions in acute cigarette smoke inhalation studies. Inhal. Toxicol. 23, 602–615 10.3109/08958378.2011.596851 21864220

[B66] van Antwerpen V. L. Theron A. J. Richards G. A. Steenkamp K. J. van der Merwe C. A. van der Walt R. Anderson R. (1995). Vitamin, E, pulmonary functions, and phagocyte-mediated oxidative stress in smokers and nonsmokers. Free Radic. Biol. Med. 18, 935–941 7797104 10.1016/0891-5849(94)00225-9

[B67] van der Strate B. W. Postma D. S. Brandsma C. A. Melgert B. N. Luinge M. A. Geerlings M. Hylkema M. N. van den Berg A. Timens W. Kerstjens H. A. (2006). Cigarette smoke-induced emphysema: a role for the B cell? Am. J. Respir. Crit. Care Med. 173, 751–758 10.1164/rccm.200504-594OC 16399994

[B68] Vanaudenaerde B. M. Verleden S. E. Vos R. de Vleeschauwer S. I. Willems-Widyastuti A. Geenens R. Van Raemdonck D. E. Dupont L. J. Verbeken E. K. Meyts I. (2011). Innate and adaptive interleukin-17-producing lymphocytes in chronic inflammatory lung disorders. Am. J. Respir. Crit. Care Med. 183, 977–986 10.1164/rccm.201007-1196PP 21097694

[B69] Vargas-Rojas M. I. Ramirez-Venegas A. Limon-Camacho L. Ochoa L. Hernandez-Zenteno R. Sansores R. H. (2011). Increase of Th17 cells in peripheral blood of patients with chronic obstructive pulmonary disease. Respir. Med. 105, 1648–1654 10.1016/j.rmed.2011.05.017 21763119

[B70] Vecchio D. Arezzini B. Pecorelli A. Valacchi G. Martorana P. A. Gardi C. (2010). Reactivity of mouse alveolar macrophages to cigarette smoke is strain dependent. Am. J. Physiol. Lung Cell. Mol. Physiol. 298, L704-L713 10.1152/ajplung.00013.2009 20154225

[B71] Vlahos R. Bozinovski S. Chan S. P. Ivanov S. Linden A. Hamilton J. A. Anderson G. P. (2010). Neutralizing granulocyte/macrophage colony-stimulating factor inhibits cigarette smoke-induced lung inflammation. Am. J. Respir. Crit. Care Med. 182, 34–40 10.1164/rccm.200912-1794OC 20203243

[B72] Witschi H. Espiritu I. Ly M. Uyeminami D. (2005). The chemopreventive effects of orally administered dexamethasone in Strain A/J mice following cessation of smoke exposure. Inhal. Toxicol. 17, 119–122 10.1080/08958370590899712 15764489

[B73] Xiao J. Wang K. Feng Y. L. Chen X. R. Xu D. Zhang M. K. (2011). Role of extracellular signal-regulated kinase 1/2 in cigarette smoke-induced mucus hypersecretion in a rat model. Chin. Med. J. (Engl.) 124, 3327–3333 22088530

[B74] Xiong Z. Leme A. S. Ray P. Shapiro S. D. Lee J. S. (2011). CX3CR1+ lung mononuclear phagocytes spatially confined to the interstitium produce TNF-alpha and IL-6 and promote cigarette smoke-induced emphysema. J. Immunol. 186, 3206–3214 10.4049/jimmunol.1003221 21278339 PMC3912553

[B75] Young R. P. Hopkins R. J. Christmas T. Black P. N. Metcalf P. Gamble G. D. (2009). COPD prevalence is increased in lung cancer, independent of age, sex and smoking history. Eur. Respir. J. 34, 380–386 10.1183/09031936.00144208 19196816

[B76] Zhao J. Harper R. Barchowsky A. Di Y. P. (2007). Identification of multiple MAPK-mediated transcription factors regulated by tobacco smoke in airway epithelial cells. Am. J. Physiol. Lung Cell. Mol. Physiol. 293, L480–L490 10.1152/ajplung.00345.2006 17496060 PMC3287033

[B77] Zhou L. Chong M. M. Littman D. R. (2009). Plasticity of CD4+ T cell lineage differentiation. Immunity 30, 646–655 10.1016/j.immuni.2009.05.001 19464987

